# Psychosocial and psychiatric factors preceding death by suicide: A case–control psychological autopsy study involving multiple data sources

**DOI:** 10.1111/sltb.12900

**Published:** 2022-07-11

**Authors:** Elaine M. McMahon, Birgit A. Greiner, Paul Corcoran, Celine Larkin, Sara Leitao, Jacklyn McCarthy, Eugene Cassidy, Colin Bradley, Carmel McAuliffe, Eve Griffin, Eileen Williamson, Tom Foster, John Gallagher, Ivan J. Perry, Nav Kapur, Ella Arensman

**Affiliations:** ^1^ National Suicide Research Foundation Cork Ireland; ^2^ School of Public Health University College Cork Cork Ireland; ^3^ National Perinatal Epidemiology Centre University College Cork Cork Ireland; ^4^ Department of Emergency Medicine University of Massachusetts Medical School Worcester Massachusetts USA; ^5^ Department of Psychiatry and Neuro‐behavioural Science University College Cork Cork Ireland; ^6^ Department of General Practice University College Cork Cork Ireland; ^7^ Southern Health and Social Care Trust Northern Ireland UK; ^8^ Centre for Mental Health and Safety University of Manchester Manchester UK; ^9^ Greater Manchester Mental Health NHS Foundation Trust Manchester UK; ^10^ NIHR Greater Manchester Patient Safety Translational Research Centre University of Manchester Manchester UK; ^11^ Australian Institute for Suicide Research and Prevention School of Applied Psychology, Griffith University Brisbane Queensland Australia

**Keywords:** case‐control, primary care, psychological autopsy, suicide

## Abstract

**Background:**

A range of factors including mental disorders and adverse life events can increase the risk of suicide. The objectives of this study were to examine psychosocial and psychiatric factors and service engagement among suicide decedents compared with living controls.

**Methods:**

A case–control study using multiple sources was conducted. Information on 132 consecutive cases of suicide was drawn from coronial files, and interviews were carried out with 35 family informants and 53 living controls. GPs completed questionnaires for 60 suicide cases and 27 controls.

**Results:**

The majority (83.3%) of suicide decedents had contacted a GP in the year prior to death, while 23.3% had 10 or more consultations during the year prior to death. Half of suicide decedents had a history of self‐harm. Suicide cases were significantly more likely than controls to have a psychiatric diagnosis (60% vs. 18.5%) and a depressive illness (36.7% vs. 14.8%). Over one‐quarter of suicide decedents had been treated as a psychiatric inpatient.

**Discussion:**

Primary care providers should be supported to deliver multidisciplinary interventions to engage, assess, and treat patients at risk of suicide, targeting those who present very frequently, those with a history of self‐harm or substance misuse, and those with psychological presentations.

## INTRODUCTION

Suicide is a major global health concern, with over 700,000 people around the world dying by suicide every year (WHO, 2021). The causes of suicide are complex, encompassing individual factors such as genetic influences and mental disorders, contextual factors such as family influences, history of abuse, socioeconomic conditions, exposure to suicidal behavior by others, access to means for a suicidal act, and lack of support in a crisis (Hawton & Pirkis, 2017). Stress‐diathesis evidence‐based models emphasize an interaction between acute stressors and suicide‐related traits or pre‐existing vulnerability (Mann & Rizk, 2020; O'Connor & Nock, 2014).

A diagnosis of mental disorder is one of the strongest risk factors for suicide (Turecki et al., 2019). However, a systematic review of psychological autopsy studies from 2000 onward found that in research papers assessing Axis I and Axis II disorders, 37.1% of suicide cases did not seem to have any mental disorder (Milner et al., 2013). While this may reflect cultural influences, under‐diagnosis of mental disorders and masked mental health conditions, it points to the need to identify risk factors other than psychopathology to guide the development of comprehensive evidence‐based suicide prevention measures.

Psychological autopsy studies deploy a systematic methodology to enhance understanding of the psychological and contextual circumstances preceding suicide (Conner et al., 2011). They are designed to provide more detailed information about risk and protective factors for suicide than only comparing the medical records of those who have died by suicide with the records of controls. Case–control psychological autopsy studies are the research standard for the quantitative study of ongoing or recent risk factors for suicide (Conner et al., 2021; Isometsä, 2001). In such studies, researchers interview proxy informants well‐known to suicide decedents shortly after death, in addition to obtaining information from healthcare professionals of the deceased and records (coronial and health care). The current study aims to examine a broad range of risk factors and protective factors for suicide due to its unique design of integrating different data sources (primary care practitioners, coroners, and family informants). The objectives were to compare psychosocial variables, psychiatric factors, and health service use among suicide cases with living controls.

## METHODS

### Design and setting

The case–control design compares suicide cases with living controls. Suicide cases were identified through coroners' records of consecutive inquests of suicides or probable suicides in the Cork City and County region in Ireland between June 2014 and September 2017. Control participants were recruited from the same general practitioner (GP) practices that suicide decedents were registered with, to control for GP practice variation and socioeconomic variables, and were frequency‐matched for age groups and gender. GP patients were chosen as the control group allowing for a group that resembled the population of the deceased and to allow matching on latent variables.

In keeping with the psychological autopsy approach, data from multiple sources were accessed for the suicide cases and controls: coronial records (suicide cases only), family informant interviews (suicide cases only), GP questionnaires (both suicide cases and controls), and self‐report interviews (controls only). Full details of the study methodology have been published previously (Arensman et al., 2019).

### Suicide case identification (coronial records)

Consecutive cases of suicide and probable suicide were identified through coroners' registration of deaths in a defined region (Cork City and County, Ireland) between June 2014 and September 2017. Inclusion criteria comprised: (a) the death having occurred within the Cork City and County coroners' defined catchment areas, (b) the verdict at conclusion of inquest being that of either “suicide” or “open,” or a narrative verdict in which the death was likely to have been a suicide, and (c) the death occurring and having gone to inquest within the timescale of the study. Suicide verdicts are returned by coroners when it has been established beyond a reasonable doubt that a person has taken his/her own life. In order to be considered a probable suicide, the death must have been self‐inflicted with evidence to indicate that the deceased intended to cause his/her death (Rosenberg et al., 1988).

There were three coroners operating in Cork City and County at the time of the study. A researcher visited the offices of the coroners every 2–4 weeks to review deaths that had gone to inquest and had been returned with a verdict of suicide, narrative verdict, or open verdict. Each coronial file contained a report of verdict from the inquest; a police summary of events; statements to police by a range of family members, friends, eyewitnesses, police, healthcare professionals, or others involved with the case; and the post‐mortem report including toxicology. Some files also included, where relevant, photographs of the scene of death and medical case notes. All documents were reviewed by the researcher using a data extraction template with the following items: date of birth, marital status, occupation, cause of death, mental health problems, precipitant stressors, and history of self‐harm.

### Control group

GP controls were recruited from the same general practices that suicide decedents attended, to control for GP practice variation, and were frequency‐matched for age and gender. GP patients were chosen as the control group allowing for a group that resembles the general population and to allow matching on latent variables. By approaching individuals with the support of their GP, we expected to minimize the self‐selection bias that can arise when recruiting from the general population. Five potential participants were randomly selected from within the given 5‐year age band and gender using the GP patient list; if the first selected patient was not contactable or unwilling to participate, the next patient was contacted and so on. Included participants were selected patients from GP practices in Cork City and County who were older than 14 years of age. Exclusion criteria for control group participants and informants were history of high‐risk self‐harm, being aged younger than 14 years, if contact was deemed to pose a risk to the safety of the researcher or the informant, or where the information obtained was unreliable due to cognitive impairment or severe mental illness.

### Interviews with family informants of suicide decedents and controls

Structured interviews with family informants of suicide cases and with controls were conducted by trained interviewers. For the suicide arm, family informants were invited to participate in an interview in the weeks following the inquest. In the first instance, a letter was sent to the next of kin using details obtained from coronial files. A follow‐up telephone call by the researcher 10 days later focused on facilitation of bereavement support, followed by an invitation to take part in an interview at home or at a research office. To be included as a family informant, the person had to be over the age of 14 years and be sufficiently acquainted with the deceased to provide rich information about the deceased's life. Controls were contacted by telephone by the research team.

The interview covered a wide range of topics including the circumstances of suicide (suicide cases only); work‐related and psychological variables (suicide cases and controls). The current analysis focused on adverse life events in the past 12 months (Brugha et al., 1985), history of self‐harm, family and personal history, psychiatric history, physical health, and alcohol/drug misuse. Adverse events examined included financial problems, legal problems, and experience of humiliation.

### GP questionnaire

As a further source of information, a questionnaire was posted to GPs, for both suicide cases and controls. This questionnaire covered the following variables: description of manner and cause of death (suicide cases only), precipitants to death (stressful and traumatic events), history of self‐harm, family/personal history, psychiatric history including psychiatric diagnoses, physical health, alcohol and drug misuse, primary care service use history, and psychiatric treatment. A researcher followed up by telephone with the GPs to respond to any of their concerns or questions.

Coroner data, informant interview data, and health professional questionnaire data were entered and analyzed using SPSS 27.

Suicide and control groups were compared in terms of proportions with each risk factor using chi‐square tests.

### Ethics

The present study received approval from the Clinical Research Ethics Committee of the Cork University Teaching Hospitals. The procedures, progress, and quality were overseen by a multidisciplinary steering group and an advisory group.

## RESULTS

### Suicide cases

One hundred and thirty‐two consecutive cases of suicide and probable suicide were identified through coroners' registration of deaths in the study region between June 2014 and September 2017, and data on relevant variables were collected from coronial files. In the case of 35 suicide cases, family informants were successfully recruited and completed the interview (26.5% response rate). There were several reasons for lower informant recruitment than expected. One of the three coroners in the study region did not give consent for the research team to approach family members regarding participation in the study. For these suicide cases (*n* = 49), the coronial files were still reviewed but it was not possible to interview a family informant, and a control participant was not sought. Other reasons why no family informant was recruited for suicide cases were as follows: potential family informant was uncontactable (*n* = 17); family informant refusal upon invitation (*n* = 25); or no next of kin details in coronial files (*n* = 8). GPs completed questionnaires for 60 of the suicide decedents (45.5% response rate).

### Control group

Recruitment of matched controls from the GP practices of the suicide cases resulted in 53 controls who completed the research interview (40.2% response rate). Reasons for the lower final number of controls than expected included: suicide cases had no GP on file; GP refusal to participate; the possibility of a limited number of potential participants for a given demographic profile; GP unwilling or unable to identify potential controls; potential participants' refusal upon invitation. Strategies to maximize recruitment included GP contact with potential controls, letter, and telephone follow‐up by the research team. The GP completed a questionnaire for 27 of the control group (50.9%).

The age range of the suicide cases was 15 to 83, with a mean age of 43 years. Of the suicide cases, 110 (83.3%) were male and 117 (88.6%) were of Irish nationality (Table 1). Over half (53.8%) were single, while 28.8% were married or cohabiting. The most common living arrangements among the suicide decedents were living alone (26.5%), followed by living with partner/spouse and children (14.4%). Of the suicide cases, 31.8% were in paid employment and 29.5% were unemployed.

**TABLE 1:** Sociodemographic characteristics of suicide decedents and control group

	Suicide (*n* = 132) (based on coroner data)		Control (n = 53) (based on self‐report interview)		
	Frequency	Percent	Frequency	Percent	*p*‐value
Age group					
15–34 years	41	31.1	14	26.4	0.82
35–49 years	39	29.5	17	32.1	
50+ years	52	39.4	22	41.5	
Sex					
Male	110	83.3	38	71.7	0.074
Female	22	16.7	15	28.3	
Nationality					
Irish	117	88.6	46	86.8	0.626
Other	14	10.6	7	13.2	
Unknown	1	0.8	0	0	
Marital status					
Single	71	53.8	13	24.5	0.0003
Widowed	5	3.8	2	3.8	0.996
Divorced/separated	15	11.4	2	3.8	0.106
Married/cohabiting	38	28.8	36	67.9	<0.0005
Unknown	3	2.3	0	0	
Living arrangements					
Alone	35	26.5	7	13.2	0.681
With family of origin	29	22	5	9.4	0.046
With partner/spouse only	13	9.8	8	15.1	0.309
With partner/spouse and children	19	14.4	27	50.9	<0.0005
With children only	5	3.8	1	1.9	0.514
Other shared (e.g., friends)	14	10.6	4	7.5	0.525
Other/unknown	17	12.9	1	1.9	0.022
Employment Status					
Paid employment (full‐time or part‐time)	42	31.8	28	52.8	0.007
Unemployed	39	29.5	3	5.7	<0.0005
Self‐employed	3	2.3	8	15.1	0.0008
Homemaker	5	3.8	1	1.9	0.509
Full‐time student	8	6.1	2	3.8	0.664
Long‐term disability	1	0.8	6	11.3	0.0006
Retired	15	11.4	4	7.5	0.439
Other/unknown	19	14.4	1	1.9	0.013

The age range of the controls was 23–80, with a mean age of 46 years. Of the controls, the majority (71.7%) were male and 86.8% were of Irish nationality. Over two‐thirds (67.9%) of control group participants were married or cohabiting, while just under a quarter (24.5%) were single. Just over half (50.9%) were living with partner/spouse and children, and the most frequently reported employment status was paid employment, reported by 28 (52.8%) of control participants.

Suicide cases and controls were compared on the prevalence of a range of adverse life events, substance use, and stressors, using data from family informant interviews for the suicide cases (n = 35) and interviews with the control group participants (*n* = 53; Table 2). Suicide decedents were significantly more likely to have a history of violent behavior (45.7%) than the controls (22.6%; *p* = 0.015) and were also significantly more likely to have experienced legal troubles than controls (31.4% of suicide cases, 11.3% of controls; *p* = 0.014). The prevalence of other traumatic events and stressors was also higher among suicide cases than controls, but differences did not reach statistical significance. Among suicide cases, a history of physical, sexual, or emotional abuse was reported for 40.0% of suicide cases, compared with 34.0% of controls. Major financial difficulties were reported for 28.6% of suicide cases and 24.5% of controls. Of the suicide cases, 31.4% had a history of alcohol misuse, compared with 17.0% of control participants, while drug misuse was reported in 22.9% of suicide decedents and 9.4% of controls.

**TABLE 2:** History of substance use, traumatic events, and stressors among suicide cases and controls

	Suicide cases (Family informant; *n* = 35)		Control group (Self‐report; *n* = 53)		*p*‐value
	Frequency	Percent	Frequency	Percent	
History of physical, sexual, or emotional abuse	14	40.0	18	34.0	0.522
History of violent behavior	16	45.7	12	22.6	0.015
History of alcohol misuse	11	31.4	9	17.0	0.089
Attempt to stop abusing alcohol (past year)	5	14.3	3	5.7	0.149
Recent increase in misuse of alcohol	3	8.6	1	1.9	0.128
History of drug misuse	8	22.9	5	9.4	0.067
Attempt to stop abusing drugs (recent)	4	11.4	2	3.8	0.147
Recent increase in misuse of drugs	2	5.7	0	0.0	‐
Legal troubles	11	31.4	6	11.3	0.014
Major financial difficulties	10	28.6	13	24.5	0.591
Humiliation (among family/ friends/workplace/online/local or national scandal)	11	31.4	20	37.7	0.632

Suicide decedents and controls were compared regarding service use and psychiatric diagnoses based on GP questionnaires for 60 suicide cases and 27 control participants (Table 3). Of the suicide decedents, 83.3% had contacted a GP at least once (51.6% at least four times) in the year prior to death, compared with 88.9% of control participants at least once (48.1% at least four times). Suicide cases were more likely to have very high rates of GP consultation, with 14 (23.3%) having contacted a GP 10 times or more in the previous year, compared with one of the controls (3.7%).

**TABLE 3:** Primary care service use and diagnoses among suicide cases and controls

	Suicide cases (GP Questionnaire, *n* = 60)		Control (GP Questionnaire, *n* = 27)	
	Frequency	Percent	Frequency	Percent
Number of times GP contacted in previous 12 months				
Contacted at least once	50	83.3	24	88.9
Contacted 1–3 times	19	31.7	11	40.7
Contacted 4–9 times	17	28.3	12	44.4
Contacted 10 times or more	14	23.3	1	3.7
Chronic physical illness	21	35.0	9	33.3
On prescribed medication for physical illness	20	33.3	16	59.3
Reason for last contact with GP				
Physical	31	51.7	25	92.6
Psychological	17	28.3	0	0.0
Both physical and psychological	7	11.7	1	3.7
Prior self‐harm	30	50.0	0	0.0
Treated as psychiatric inpatient	16	26.7	1	3.7
Prescribed psychotropic medication by GP	29	48.3	4	14.8
Prescribed psychotropic medication by a psychiatrist	11	18.3	2	7.4
Psychiatric diagnosis (primary and secondary diagnoses recorded in 15 suicide cases)				
None recorded	24	40.0	22	81.5
Depressive illness	22	36.7	4	14.8
Alcohol misuse/dependence	5	8.3	0	0.0
Bipolar affective disorder	2	3.3	0	0.0
Anxiety, phobia, panic disorder	7	11.7	1	3.7
Personality disorder	4	6.7	0	0.0
Schizophrenia or other psychotic disorder	1	1.7	0	0.0
Psychiatric illness, other/unspecified diagnosis	9	15.0	0	0.0
Adjustment disorder	1	1.7	0	0.0

Chronic physical illness was reported for similar proportions of the suicide and control groups (35.0% of suicide cases, 33.3% of controls). However, controls were more likely to be on prescribed medication for physical illness (33.3% of suicide cases, 59.3% of controls). Suicide cases were more likely to have had a psychological reason for their last GP consultation than controls (28.3% of suicide cases, none of the control group). Prior self‐harm was reported by the GP for 50.0% of the suicide decedents, compared with none of the control group. The majority of suicide cases had at least one diagnosed psychiatric disorder, with a higher prevalence than the control group (60.0% of suicide cases and 18.5% of controls). Depressive illness was the most common psychiatric diagnosis for both study groups, which was recorded for 36.7% of suicide cases and 14.8% of controls. Anxiety, phobia, or panic disorder was also more common among suicide decedents, with this diagnosis recorded for 11.7% of suicide cases and 3.7% of controls. A diagnosis of alcohol misuse or dependence was noted for 8.3% of suicide cases and none of the control group. A smaller number of suicide cases had a diagnosis of bipolar affective disorder, personality disorder, schizophrenia/other psychotic disorder, and adjustment disorder, while none of the controls had received any of these diagnoses. Over one‐quarter (26.7%) of suicide decedents had a prior psychiatric admission, which was higher than the control group (3.7%). Of the suicide cases, 48.3% had been prescribed psychotropic medication by their GP, compared with 14.8% of the control group.

## DISCUSSION

In this study, we have identified the sociodemographic profile of those who have died by suicide as well as potential precipitant triggers and prior service use. Interviews with family informants provided an in‐depth examination of psychiatric history, antecedent life events, and psychosocial stressors preceding suicide and allowed for a comparison with living controls recruited from the same GP practices as the suicide cases. The inclusion of GP questionnaires provided valuable insight into health service use in the period prior to death.

The suicide cases were predominantly male and were more likely than controls to be single, living alone, and unemployed. Compared with controls, suicide decedents were more likely to have a history of violent behavior and to have had recent legal troubles. High rates of alcohol and drug misuse were also seen in the suicide cases.

The findings of the present study are consistent with evidence from major psychological autopsy studies that nearly all suicides have experienced at least one (usually more) adverse life event within 1 year of death, concentrated in the last few months (Foster et al., 1997). Controlled studies have revealed specific adverse life events, notably interpersonal conflict, as risk factors for suicide and some evidence of a dose–response effect. Adverse life events may precipitate and exacerbate psychiatric disorders and/or increase the desire to die in vulnerable people (Foster et al., 2011). An unexpected finding was the similar proportion of the suicide cases and controls for whom a history of physical, emotional, or sexual abuse was reported. High rates of abuse history were expected among suicide cases due to established associations with suicidal behavior (Troya et al., 2021). It may be the case that informant reporting of abuse history for suicide decedents is subject to recall, reluctance to report sensitive issues, or other bias.

In our study, one‐third of suicide decedents had a history of alcohol misuse, as reported by family informants, with almost double the prevalence of alcohol misuse among suicide cases than controls. Interestingly, GP‐reported prevalence of alcohol misuse/dependence among suicide cases was much lower (8.3%). The prevalence of alcohol misuse/dependence in the sole psychological autopsy study of suicide in Northern Ireland was 43% (Foster et al., 1997). An Irish study of GP patient records has highlighted a lack of documentation of alcohol problems and a need to reinforce positive attitudes among GPs regarding preventive work (O'Regan et al., 2018). Internationally, previous psychological autopsy studies have reported a prevalence of alcohol misuse among suicide decedents of between 0% (Khan et al., 2008) and 61% (Kõlves et al., 2006). In the Estonian study, the highest prevalence of alcohol misuse among suicide decedents was in middle‐aged men (Kõlves et al., 2006). Alcohol misuse can increase the risk of completed suicide through its short‐term (impulse control, dysphoria, reduced problem‐solving, and compounding lethality) and long‐term (comorbid psychiatric disorder, psychosocial adversity, and physical health complications) effects (Borges et al., 2017). As people with alcohol use disorders who are at elevated risk of suicide frequently have other mental disorders, notably depressive disorders, and relationship difficulties that also increase suicide risk, interventions are needed to address such concomitant stressors. Previous research has reported that, among suicide attempters with mood disorders, a history of lifetime substance use disorder was associated with more frequent and more lethal suicide attempts (Rizk et al., 2021). Population‐level interventions to restrict access to alcohol may have an impact on suicide rates (Xuan et al., 2016).

In this study, 40% of suicide decedents had no psychiatric diagnosis. This is a similar figure to the 37% cited in Milner et al.’s international review (Milner et al., 2013). Unidentified, masked, and untreated depression (and other mental disorders) may contribute to some suicides, highlighting the important role of primary care professionals in diagnosing and treating such disorders. A recent review has reported evidence that training primary care doctors in depression recognition and treatment prevents suicide. The review also reported the efficacy of antidepressants, cognitive‐behavioral therapy, and dialectical behavior therapy in preventing suicidal behavior (Mann et al., 2021). Several large‐scale community studies have shown that education of GPs and other medical professionals on the recognition and appropriate pharmacotherapy of depression, particularly in combination with psychosocial interventions and public education, significantly improves identification and treatment of depression and consequentially reduces the rate of completed and attempted suicide (Rihmer et al., 2012).

While we have identified key risk factors for suicide, it is difficult to examine the relative significance of the various domains, or to identify cumulative effects of adverse events, precipitants, and mental disorders. A psychological autopsy study from Bangladesh, which estimated the population attributable fraction for key clinical and social risk factors for suicide, found that life events were responsible for the largest proportion of suicide deaths, followed by mental disorder, sexual abuse, and social isolation (Arafat et al., 2021). The influence of gender, age, and culture on the relative contributions of adversity and mental disorder to suicide warrant further research.

The vast majority of suicide decedents in this study had consulted a GP at least once in the year prior to death. The proportion (83.3%) was close to the average figure revealed in a systematic review of the literature from 2000 to 2017 (80% in previous year, 44% in previous month, 16% in previous week) (Stene‐Larsen & Reneflot, 2019), but slightly lower than the proportions in a major psychological autopsy study of suicide in Northern Ireland (90% in previous year, 34% in previous month, and 14% in previous week) (Foster et al., 1997) and a large‐scale French study (89% in previous year, 46% in previous month, and 21% in previous week) (Laanani et al., 2020). A review of research on service use prior to suicide reported a consistent finding that contact with primary health care among suicide cases was highest in the year prior to suicide (Stene‐Larsen & Reneflot, 2019). In a recent Dutch study that considered the 1‐ to 2‐year period before suicide, 52% of the suicide decedents received care from mental health services, 41% received GP care only, and 7% received neither (Elzinga et al., 2021). Half of suicide decedents in our study had a history of self‐harm of which their GP was aware, highlighting opportunities for timely intervention and highlighting the importance of the therapeutic alliance between GP and patient. People who present very frequently to primary care are a target for multidisciplinary intervention. Studies examining GPs' experiences of managing suicidal patients have identified barriers including a potential lack of confidence in the management of suicidal patients, structural inadequacies in mental health service provision (Leavey et al., 2017), and difficulties in assessing suicide risk (Elzinga et al., 2020).

### Strengths and limitations

While this study has successfully identified and examined a large number of consecutive suicide cases from coroners' records, it proved difficult to recruit the original sample size target of family informants of suicide cases and controls for in‐depth interview and case–control comparison (Arensman et al., 2019). Issues which arose included the withdrawal of consent to approach family informants by one of the three coroners in the region, affecting over one‐third of the suicide cases. Additionally, difficulties in contacting next of kin affected recruitment, while a relatively small number of family members declined to participate when approached. Recruitment of the control group was carried out via GPs of the suicide decedents, and so was dependent on the provision of details of each suicide case's GP within the coronial files. This information was not available in a large number of cases. Control recruitment also depended upon the GP agreeing to the identification and invitation of potential controls from their patient list, with matching by age and gender to the suicide case. While the aim was that the matching of cases and controls led to groups that were comparable on latent variables, in some cases, it was not possible to recruit both a family informant and directly matched control participant based on a particular suicide case. Notwithstanding these issues, the recruitment of 53 controls was a positive feature of this study, considering the methodology involved. Completion of the GP questionnaire for cases and controls was also not maximized, reflecting the demands on time and resources and ongoing stigma related to suicide in primary care.

Family informants are an essential element of the psychological autopsy approach. However, challenges with recruitment of bereaved family members described here mean that it may be difficult to recruit informants for a representative sample of suicide cases. In the case of the control group, those with a history of high‐risk self‐harm or serious mental illness were excluded, which may have led to a control group, which was not representative of the primary care population from which it was drawn. Systematic recall bias on the part of informants is also possible. The reliability of the information obtained from various sources in psychological autopsy studies, and how these are reconciled in the absence of self‐report, is an ongoing methodological issue that requires further testing of empirical data. Validity studies have reported acceptable validity for the reporting of life events by informants (Mo et al., 2019; Zhang et al., 2003). There may also be a tendency among bereaved informants to disproportionately recall adverse events that might help them to make sense of suicide, admittedly a potential limitation of the use of living controls in psychological autopsy studies. In this study, psychiatric diagnoses were not made according to an official diagnostic classification system and were based on GP questionnaires only.

Notwithstanding these limitations, this study successfully employed multiple data sources, allowing for the assessment of a range of psychosocial and psychiatric risk factors for suicide. We have used coroner data to identify a large cohort of consecutive suicide cases and recruited family informants and a representative control group drawn from the same GP practices as the suicide cases. A strength of the study was the investigation of consecutive cases of suicide which reduces selection bias. The standardized approach taken in relation to the methods and procedures, including specialized and consistent training and supervision of interviewers, was also a strength of this study.

## CONCLUSION

The sociodemographic and psychiatric factors identified, as well as the importance of proximal triggers and adverse life events, can aid in the identification of those at greatest risk. Primary care providers should be resourced and supported to deliver multidisciplinary intervention to engage, assess, and treat patients in primary care at risk of suicide. Our findings suggest targeting primary care patients who present very frequently, those with a history of self‐harm or substance misuse, those with psychological presentations, and those who have physical symptoms not requiring medication. If recruitment challenges can be overcome, the case–control psychological autopsy approach involving multiple data source provides valuable insights to develop suicide prevention strategies.

## CONFLICT OF INTEREST

The authors have declared that no competing interests exist.

## PATIENT CONSENT STATEMENT

The study participants signed a written consent form, which was explained in detail to them before taking part in the study.

## ETHICAL APPROVAL

Ethics approval was granted by the Clinical Research Ethics Committee of the Cork University Teaching Hospitals, reference: ECM 5 [6] 01/04/14.

## Data Availability

The data that support the findings of this study are available on request from the corresponding author. The data are not publicly available due to privacy or ethical restrictions.
